# Euglycemic Diabetic Ketoacidosis Triggered by Sodium-Glucose Cotransporter 2 (SGLT2) Inhibitor Use in a Patient With Type 2 Diabetes Mellitus

**DOI:** 10.7759/cureus.84170

**Published:** 2025-05-15

**Authors:** Yusuf Almascati, Ali Alhumaiqani

**Affiliations:** 1 Internal Medicine, King Hamad American Mission Hospital, A'ali, BHR

**Keywords:** diabetic ketoacidosis, dka, euglycemic diabetic ketoacidosis, high anion gap metabolic acidosis, sglt2 eudka

## Abstract

Euglycemic diabetic ketoacidosis (EuDKA) is a potentially life-threatening complication associated with sodium-glucose co-transporter 2 (SGLT2) inhibitors. Its subtle presentation, often lacking the hallmark of marked hyperglycemia, can lead to delayed recognition and treatment. We present the case of a 53-year-old female with type 2 diabetes mellitus who developed EuDKA following the abrupt discontinuation of long-term insulin therapy and transition to an SGLT2 inhibitor. Her vague symptoms and near-normal glucose levels concealed the severity of her condition, delaying the diagnosis. Once identified, she was managed with intravenous insulin, dextrose-containing fluids, and potassium replacement, resulting in resolution of acidosis and normalization of serum ketones. This case focuses on the diagnostic challenges posed by euglycemic DKA and highlights the importance of considering it in patients with SGLT2 inhibitors, even without significant hyperglycemia. It also emphasizes the need for careful clinical judgment when adjusting diabetic regimens and calls for more structured prescribing guidelines as these agents gain broader use.

## Introduction

Euglycemic diabetic ketoacidosis (EuDKA) is a recognized variant of diabetic ketoacidosis (DKA) characterized by the presence of ketoacidosis with blood glucose levels below 13.9 mmol/L (250 mg/dL) [1]. First described by Munro et al. in the 1970s [2], EuDKA has historically been considered rare, but its incidence has increased with the growing use of sodium-glucose co-transporter 2 (SGLT2) inhibitors. These agents have been widely adopted not only for their glycemic effects in type 2 diabetes mellitus (T2DM) but also for their proven cardiovascular and renal benefits in patients with heart failure and chronic kidney disease, even when used in the absence of overt hyperglycemia [3,4]. However, their use has been associated with an increased risk of DKA, particularly in its euglycemic form.

The pathophysiology of EuDKA in the setting of SGLT2 inhibitor therapy is multifactorial. Through enhanced glycosuria, they lower plasma glucose concentrations, reducing insulin secretion and promoting glucagon release. This shift favors ketogenesis and, when combined with increased renal reabsorption of ketone bodies, creates a biochemical environment that can lead to ketoacidosis without significant hyperglycemia [5,6]. In a systematic review of 34 published cases of SGLT2 inhibitor-associated DKA, Burke et al. reported that 71% met the criteria for EuDKA [7], demonstrating the extent to which this presentation can occur and how easily it may be overlooked in clinical settings.

Although DKA is often precipitated by triggers such as infection, prolonged fasting, or recent surgery, EuDKA may occur without any identifiable stressor [8]. This case describes a patient who developed EuDKA following an abrupt discontinuation of long-term insulin therapy and the initiation of an SGLT2 inhibitor, in the absence of any precipitating illness. It draws attention to the diagnostic complexity of EuDKA, particularly when conventional clinical signs are absent, and how metabolic deterioration can arise solely from medication changes.

While this presentation is increasingly recognized within endocrinology and diabetes care, many general practitioners remain unfamiliar with its atypical features. Greater clinical awareness is necessary to ensure early identification, especially in patients changing their diabetic regimens. Particular attention should be given to patients recently transitioned off insulin or started on SGLT2 inhibitors, as this subgroup may develop ketosis and metabolic acidosis without significant hyperglycemia [9]. Early evaluation with serum ketones and venous blood gas testing should be considered in such contexts to prevent progression [10]. This report emphasizes these diagnostic pitfalls and advocates for more individualized approaches when modifying diabetes treatment regimens, particularly in insulin-dependent patients.

## Case presentation

A 53-year-old female with a known history of T2DM presented to the emergency department with a primary complaint of progressive fatigue that had been worsening over one month. She also reported intermittent mild abdominal discomfort, persistent nausea, reduced appetite, and unintentional weight loss of approximately 7 kg over the preceding two months. Despite feeling generally unwell, she sought medical attention due to increasing concern over the persistence and gradual progression of her symptoms. She denied vomiting, diarrhea, dysuria, fever, or chills.

Two months prior to presentation, her longstanding basal-bolus insulin regimen was abruptly discontinued on the recommendation of a newly consulted physician. The rationale for stopping insulin was not documented. Although the patient expressed concern, she was reassured that insulin was no longer necessary. Instead, she switched to antihyperglycemic agents, including a combination of glimepiride/metformin, pioglitazone, and empagliflozin. She reported worsening nausea, which she attributed to the new oral medications, but remained adherent to the prescribed regimen.

Although the patient’s HbA1c at presentation was elevated (9.0%), there was no documentation of glycemic status when the insulin was discontinued. The decision appeared to be based solely on a shift in therapeutic approach rather than an objective reassessment of glycemic control or endogenous insulin sufficiency. No formal assessment, such as a fasting C-peptide or glucose log review, was recorded to justify the change. This lack of documentation raised concerns regarding the clinical reasoning behind withdrawing insulin in a patient with longstanding diabetes.

During the presentation, the patient was alert and oriented. Her vital signs were within normal limits: blood pressure of 126/74 mmHg, heart rate of 88 beats per minute, respiratory rate of 17 breaths per minute, oxygen saturation of 98% on room air, and temperature of 36.8 degrees Celsius. Her abdomen was soft and non-distended, with normal bowel sounds. There was mild epigastric tenderness on palpation, but no guarding, rebound tenderness, or organomegaly.

Laboratory findings testing revealed a high anion gap metabolic acidosis in the setting of near-normal blood glucose levels (Table 1). Serum and urine ketones were strongly positive. Other findings included hypokalemia, an elevated HbA1c at 9.0%, and a C-peptide level of 0.88 ng/ml. No evidence of infection, dietary restriction, recent illness, or medication non-adherence was identified. These results were consistent with a diagnosis of EuDKA.

**Table 1:** Laboratory findings on admission pH: potential of hydrogen, pCO_2_: partial pressure of carbon dioxide, HbA1c: hemoglobin A1c

Laboratory finding	Result (on admission)	Reference range
Glucose	7 mmol/L	3.9-5.5 mmol/L
Venous pH	7.30	7.35-7.45
Bicarbonate	15 mmol/L	22-29 mmol/L
Anion gap	21 mmol/L	8-12 mmol/L
pCO_2_	31 mmHg	35-45 mmHg
Potassium	3.2 mmol/L	3.5-5.2 mmol/L
Urinalysis	4+ ketones	Negative
Serum ketones	3+ (strongly positive)	Negative
HbA1c	9.0%	<7.0%
C-peptide	0.88 ng/mL	0.48-5.05 ng/mL

The patient was started on a low-dose intravenous insulin infusion protocol in combination with dextrose-containing intravenous fluids and potassium replacement. Given the presence of metabolic acidosis and ketosis despite near-normal glucose levels, the low-dose insulin protocol was selected in accordance with institutional DKA guidelines. Dextrose was administered in parallel to mitigate the risk of hypoglycemia during ongoing insulin therapy and facilitate ketone clearance.

The patient’s initial serum potassium level of 3.2 mmol/L guided potassium replacement. Given that this value was below normal values, potassium was replaced, and supplementation was titrated based on hourly serum levels to prevent worsening hypokalemia during insulin-mediated intracellular shifts. Electrolytes and capillary blood glucose were monitored hourly throughout the infusion.

**Figure 1:**
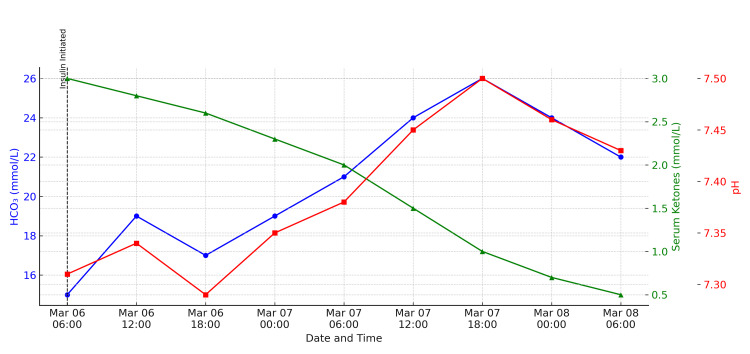
Trend of venous blood gas parameters and serum ketone levels Blue line: serum bicarbonate (HCO₃), red line: venous pH, green line: serum ketone levels

All oral antihyperglycemic agents, including the SGLT2 inhibitor, were withheld upon admission. Serial venous blood gas measurements during hospitalization demonstrated progressive correction of acidosis, improving base excess and ketone clearance (Figure 1). She showed gradual clinical improvement with resolution of acidosis and normalization of serum ketones. After stabilization, she was safely transitioned back to a basal-bolus insulin regimen. She was discharged with reinforced diabetes education, medication counseling, and scheduled follow-up with both primary care and endocrinology for ongoing glycemic management and review of long-term treatment.

## Discussion

Despite advancements in diabetes management, DKA remains a potentially life-threatening complication. While classical DKA is well recognized, EuDKA poses a unique diagnostic challenge due to the absence of significant hyperglycemia. In a review of adverse events data submitted to the FDA, Peters et al. highlighted that SGLT2 inhibitors may account for a substantial proportion of DKA cases presenting with atypical glycemic profiles, suggesting that the true incidence of EuDKA may be overlooked due to diagnostic delays and misclassification [8].

The diagnosis of EuDKA requires a high index of suspicion. Presentations are often vague, and without the typical hyperglycemia, the severity of metabolic decompensation can go unrecognized. In this case, the patient presented with nonspecific complaints of fatigue, mild abdominal discomfort, and decreased oral intake. Her blood glucose levels remained within a near-normal range, which initially masked the seriousness of her condition. The diagnosis was made only after routine biochemical evaluation revealed a high anion gap metabolic acidosis and ketonemia.

The absence of traditional DKA triggers further distinguishes this case. There was no infection, dietary restriction, recent illness, or medication non-adherence. Instead, the likely precipitant was the abrupt discontinuation of long-term insulin therapy and the initiation of an SGLT2 inhibitor. While the HbA1c on admission was markedly elevated at 9.0%, there was no documentation of glucose control at the time of medication change. No metabolic markers, such as a fasting C-peptide or serial glucose records, were reviewed before deciding to stop insulin. This highlights a critical gap in individualized treatment planning and raises concerns regarding the inappropriate de-escalation of insulin in patients with established insulin dependence.

Although SGLT2 inhibitors are generally well tolerated and offer considerable benefits in patients with T2DM, heart failure, and chronic kidney disease, their increasing use has led to a shift in prescribing patterns. These agents are sometimes introduced early or used to replace insulin, particularly in patients perceived as non-adherent or at risk for insulin-related side effects. However, such changes may have unintended consequences in individuals with impaired endogenous insulin production. In this case, the patient’s metabolic reserve was likely insufficient to counter the pro-ketogenic effects of SGLT2 inhibition.

Management of EuDKA follows the same principles as traditional DKA, with insulin administration, fluid resuscitation, and electrolyte correction. However, clinicians may be less inclined to start an insulin infusion in patients without significant hyperglycemia, especially if EuDKA is not considered early in the differential. The addition of dextrose-containing fluids alongside insulin is often necessary to prevent hypoglycemia while enabling ongoing ketone clearance. In this case, the patient responded to the management plan with progressive correction of her acid-base status and normalization of ketone levels.

This case offers two important clinical takeaways. First, it emphasizes the need for heightened vigilance for EuDKA in patients on SGLT2 inhibitors, even without classical DKA features. Second, and more uniquely, it draws attention to the increasing tendency to prescribe SGLT2 inhibitors without comprehensively evaluating glycemic control or insulin requirements. As these agents gain broader indications beyond diabetes, especially in cardiology and nephrology, a more cautious and individualized approach is essential. Treatment decisions must be grounded in objective metabolic assessment rather than enthusiasm for newer agents alone. Patient education and careful medication reconciliation cannot be overstated in preventing adverse outcomes.

## Conclusions

EuDKA is an uncommon but clinically significant presentation of a metabolic emergency that can progress rapidly if not promptly identified. Diagnosis relies on maintaining a high index of suspicion, particularly in patients receiving SGLT2 inhibitors who do not exhibit the marked hyperglycemia typically associated with DKA. This case demonstrated the potential risks of altering established insulin regimens without adequate metabolic evaluation and underscores the importance of individualized treatment planning. As the use of SGLT2 inhibitors expands across medical specialties, clinicians must remain vigilant to atypical presentations of DKA and ensure that comprehensive glycemic control and insulin dependence assessments guide prescribing decisions. EuDKA is a serious and often underrecognized complication that may occur in the absence of classical triggers or significant hyperglycemia. This case highlights how inappropriate insulin regimen alteration, without adequate glycemic control or insulin dependence assessment, can precipitate significant metabolic decompensation. It also reinforces the need for clinicians to consider EuDKA in the differential diagnosis for patients on SGLT2 inhibitors who present with nonspecific symptoms and biochemical acidosis.

The patient responded well to standard DKA management, including insulin infusion, intravenous fluids, and potassium replacement, and was successfully transitioned back to a basal-bolus regimen upon discharge. While this case demonstrates a favorable outcome, it reflects a broader concern regarding prematurely substituting newer anti-diabetic agents instead of insulin without proper clinical evaluation. It contributes to the growing body of literature emphasizing the importance of individualized treatment decisions and timely recognition of atypical DKA presentations in the context of evolving diabetes therapies. Further research and developing clearer clinical guidelines for safe SGLT2 inhibitors, particularly in patients with insulin dependence, are warranted to guide evidence-based practice.
